# MOVING TOWARD BEST PRACTICES IN STATE APPROACHES TO SUPPORTING FAMILY CAREGIVERS OF OLDER ADULTS

**DOI:** 10.1093/geroni/igz038.1418

**Published:** 2019-11-08

**Authors:** Julia Burgdorf, Jennifer Aufill, Jennifer L Wolff

**Affiliations:** 1 Johns Hopkins Bloomberg School of Public Health, Baltimore, Maryland, United States; 2 Johns Hopkins University Bloomberg School of Public Health, Baltimore, Maryland, United States; 3 Johns Hopkins University, Maryland, United States

## Abstract

In January 2018, Congress passed the Recognize, Assist, Include, Support, and Engage (RAISE) Family Caregivers Act to establish a national strategy to acknowledge and assist family caregivers. The success of such an effort will be contingent on knowledge and dissemination of best practices. We conducted a policy project funded by the Milbank Memorial Fund that profiles five geographically and politically diverse states that have pursued novel approaches to supporting family and unpaid caregivers of older adults. We discuss findings from national and state reports, state aging plans, and interviews with 26 key informants. We find that each state (Hawaii, Maine, Minnesota, Tennessee, Washington) sought to strengthen supports for family caregivers as an element of broader community-based long-term services and supports strategies. Supports for family caregivers included financial support for working caregivers, caregiver assessment and care planning, and expanded access to respite care: several states included these services within programs targeted to persons at high risk of institutionalization or Medicaid-entry. Key informants noted the importance of tailoring programs to suit each state’s unique demographic, geographic, and service delivery context, and reported that individual stories, advocacy, and data were critical to placing family caregiving on legislative policy agendas. State leaders highlighted the pivotal role of state aging networks in efforts to supporting caregivers, given these entities’ deep knowledge of local community needs and challenges and established relationships with local service providers. Meaningful change often required a long-term commitment and sustained incrementalism, typically by innovating on a smaller scale before expanding statewide.

